# No credible evidence supporting claims of the laboratory engineering of SARS-CoV-2

**DOI:** 10.1080/22221751.2020.1733440

**Published:** 2020-02-26

**Authors:** Shan-Lu Liu, Linda J. Saif, Susan R. Weiss, Lishan Su

**Affiliations:** a Center for Retrovirus Research, The Ohio State University, Columbus, OH, USA; b Department of Veterinary Biosciences, The Ohio State University, Columbus, OH, USA; c Department of Microbial Infection and Immunity, The Ohio State University, Columbus, OH, USA; d Viruses and Emerging Pathogens Program, Infectious Diseases Institute, The Ohio State University, Columbus, OH, USA; e Food Animal Health Research Program, Ohio Agricultural Research and Development Center, CFAES, Department of Veterinary Preventive Medicine, The Ohio State University, Wooster, OH, USA; f Department of Microbiology, Perelman School of Medicine, University of Pennsylvania, Philadelphia, PA, USA; g Lineberger Comprehensive Cancer Center, Department of Microbiology and Immunology, University of North Carolina at Chapel Hill, Chapel Hill, NC, USA

The emergence and outbreak of a newly discovered acute respiratory disease in Wuhan, China, has affected greater than 40,000 people, and killed more than 1,000 as of Feb. 10, 2020. A new human coronavirus, SARS-CoV-2, was quickly identified, and the associated disease is now referred to as coronavirus disease discovered in 2019 (COVID-19) (https://globalbiodefense.com/novel-coronavirus-covid-19-portal/).

According to what has been reported [1–3], COVID-2019 seems to have similar clinical manifestations to that of the severe acute respiratory syndrome (SARS) caused by SARS-CoV. The SARS-CoV-2 genome sequence also has ∼80% identity with SARS-CoV, but it is most similar to some bat beta-coronaviruses, with the highest being >96% identity [4,5].

Currently, there are speculations, rumours and conspiracy theories that SARS-CoV-2 is of laboratory origin. Some people have alleged that the human SARS-CoV-2 was leaked directly from a laboratory in Wuhan where a bat CoV (RaTG13) was recently reported, which shared ∼96% homology with the SARS-CoV-2 [4]. However, as we know, the human SARS-CoV and intermediate host palm civet SARS-like CoV shared 99.8% homology, with a total of 202 single-nucleotide (nt) variations (SNVs) identified across the genome [6]. Given that there are greater than 1,100 nt differences between the human SARS-CoV-2 and the bat RaTG13-CoV [4], which are distributed throughout the genome in a naturally occurring pattern following the evolutionary characteristics typical of CoVs, it is highly unlikely that RaTG13 CoV is the immediate source of SARS-CoV-2. The absence of a logical targeted pattern in the new viral sequences and a close relative in a wildlife species (bats) are the most revealing signs that SARS-CoV-2 evolved by natural evolution. A search for an intermediate animal host between bats and humans is needed to identify animal CoVs more closely related to human SARS-CoV-2. There is speculation that pangolins might carry CoVs closely related to SARS-CoV-2, but the data to substantiate this is not yet published (https://www.nature.com/articles/d41586-020-00364-2).

Another claim in Chinese social media points to a Nature Medicine paper published in 2015 [7], which reports the construction of a chimeric CoV with a bat CoV S gene (SHC014) in the backbone of a SARS CoV that has adapted to infect mice (MA15) and is capable of infecting human cells [8]. However, this claim lacks any scientific basis and must be discounted because of significant divergence in the genetic sequence of this construct with the new SARS-CoV-2 (>5,000 nucleotides).

The mouse-adapted SARS virus (MA15) [9] was generated by serial passage of an infectious wildtype SARS CoV clone in the respiratory tract of BALB/c mice. After 15 passages in mice, the SARS-CoV gained elevated replication and lung pathogenesis in aged mice (hence M15), due to six coding genetic mutations associated with mouse adaptation. It is likely that MA15 is highly attenuated to replicate in human cells or patients due to the mouse adaptation.

It was proposed that the S gene from bat-derived CoV, unlike that from human patients- or civets-derived viruses, was unable to use human ACE2 as a receptor for entry into human cells [10,11]. Civets were proposed to be an intermediate host of the bat-CoVs, capable of spreading SARS CoV to humans [6,12]. However, in 2013 several novel bat coronaviruses were isolated from Chinese horseshoe bats and the bat SARS-like or SL-CoV-WIV1 was able to use ACE2 from humans, civets and Chinese horseshoe bats for entry [8]. Combined with evolutionary evidence that the bat ACE2 gene has been positively selected at the same contact sites as the human ACE2 gene for interacting with SARS CoV [13], it was proposed that an intermediate host may not be necessary and that some bat SL-CoVs may be able to directly infect human hosts. To directly address this possibility, the exact S gene from bat coronavirus SL-SHC014 was synthesized and used to generate a chimeric virus in the mouse adapted MA15 SARS-CoV backbone. The resultant SL-SHC014-MA15 virus could indeed efficiently use human ACE2 and replicate in primary human airway cells to similar titres as epidemic strains of SARS-CoV. While SL-SHC014-MA15 can replicate efficiently in young and aged mouse lungs, infection was attenuated, and less virus antigen was present in the airway epithelium as compared to SARS MA15, which causes lethal outcomes in aged mice [7].

Due to the elevated pathogenic activity of the SHC014-MA15 chimeric virus relative to MA15 chimeric virus with the original human SARS S gene in mice, such experiments with SL-SHC014-MA15 chimeric virus were later restricted as gain of function (GOF) studies under the US government-mandated pause policy (https://www.nih.gov/about-nih/who-we-are/nih-director/statements/nih-lifts-funding-pause-gain-function-research). The current COVID-2019 epidemic has restarted the debate over the risks of constructing such viruses that could have pandemic potential, irrespective of the finding that these bat CoVs already exist in nature. Regardless, upon careful phylogenetic analyses by multiple international groups [5,14], the SARS-CoV-2 is undoubtedly distinct from SL-SHC014-MA15, with >6,000 nucleotide differences across the whole genome. Therefore, once again there is no credible evidence to support the claim that the SARS-CoV-2 is derived from the chimeric SL-SHC014-MA15 virus.

There are also rumours that the SARS-CoV-2 was artificially, or intentionally, made by humans in the lab, and this is highlighted in one manuscript submitted to BioRxiv (a manuscript sharing site prior to any peer review), claiming that SARS-CoV-2 has HIV sequence in it and was thus likely generated in the laboratory. In a rebuttal paper led by an HIV-1 virologist Dr. Feng Gao, they used careful bioinformatics analyses to demonstrate that the original claim of multiple HIV insertions into the SARS-CoV-2 is not HIV-1 specific but random [15]. Because of the many concerns raised by the international community, the authors who made the initial claim have already withdrawn this report.

Evolution is stepwise and accrues mutations gradually over time, whereas synthetic constructs would typically use a known backbone and introduce logical or targeted changes instead of the randomly occurring mutations that are present in naturally isolated viruses such as bat CoV RaTG13. In our view, there is currently no credible evidence to support the claim that SARS-CoV-2 originated from a laboratory-engineered CoV. It is more likely that SARS-CoV-2 is a recombinant CoV generated in nature between a bat CoV and another coronavirus in an intermediate animal host. More studies are needed to explore this possibility and resolve the natural origin of SARS-CoV-2. We should emphasize that, although SARS-CoV-2 shows no evidence of laboratory origin, viruses with such great public health threats must be handled properly in the laboratory and also properly regulated by the scientific community and governments.
